# Acyl Chain Specificity of Marine *Streptomyces klenkii* PhosPholipase D and Its Application in Enzymatic Preparation of Phosphatidylserine

**DOI:** 10.3390/ijms221910580

**Published:** 2021-09-30

**Authors:** Rongkang Hu, Ruiguo Cui, Dongming Lan, Fanghua Wang, Yonghua Wang

**Affiliations:** School of Food Science and Engineering, South China University of Technology, Guangzhou 510640, China; 201810106652@mail.scut.edu.cn (R.H.); 201810106665@mail.scut.edu.cn (R.C.); dmlan@scut.edu.cn (D.L.)

**Keywords:** phospholipase D, enzyme characterization, chain length specificity, molecular docking, phosphatidylserine

## Abstract

Mining of phospholipase D (PLD) with altered acyl group recognition except its head group specificity is also useful in terms of specific acyl size phospholipid production and as diagnostic reagents for quantifying specific phospholipid species. Microbial PLDs from *Actinomycetes*, especially *Streptomyces*, best fit this process requirements. In the present studies, a new PLD from marine *Streptomyces klenkii* (SkPLD) was purified and biochemically characterized. The optimal reaction temperature and pH of SkPLD were determined to be 60 °C and 8.0, respectively. Kinetic analysis showed that SkPLD had the relatively high catalytic efficiency toward phosphatidylcholines (PCs) with medium acyl chain length, especially 12:0/12:0-PC (67.13 S^−1^ mM^−1^), but lower catalytic efficiency toward PCs with long acyl chain (>16 fatty acids). Molecular docking results indicated that the different catalytic efficiency was related to the increased steric hindrance of long acyl-chains in the substrate-binding pockets and differences in hydrogen-bond interactions between the acyl chains and substrate-binding pockets. The enzyme displayed suitable transphosphatidylation activity and the reaction process showed 26.18% yield with L-serine and soybean PC as substrates. Present study not only enriched the PLD enzyme library but also provide guidance for the further mining of PLDs with special phospholipids recognition properties.

## 1. Introduction

Phospholipids (PLs) are one kinds of mixed lipids containing phosphoric acid, which are the basic component of cell membrane and the essential substance of life [1]. PLs have been broadly applied in emulsifiers, components of cosmetics, medical formulations and for liposome preparations because of their unique chemical structure and healthcare functions [2]. In recent years, due to the increasing cost of healthcare-related products and expanding health benefits of functional lipids, there is increasing interest in PLs with varying molecular properties (such as charge, polarity, size, etc.) to obtain special functional PLs with excellent process properties and outstanding physiological and pharmacological functions [3]. Enzymatic modification of natural PLs has become an important way to realize the high value utilization of PLs.

PLs consist of a glycerol backbone and a polar head group located at the *sn*-3 position of the glycerol backbone. Phospholipase D (PLD, EC 3.1.4.4) hydrolyze the phosphodiester bond between phosphate at the *sn*-3 position of PL and the polar head groups. PLD-catalyzed transphosphatidylation is especially useful in synthesis of naturally less abundant PLs such as phosphatidylglycerol (PG), phosphatidylethanolamine (PE), phosphatidylserine (PS), and phosphatidylinositol (PI) by exchanging choline head group of phosphatidylcholine (PC) [4,5,6,7]. In addition, PLD with altered acyl group recognition except its head group specificity is also useful for the production of specific PL products and as diagnostic reagents for quantifying specific PL species [8]. PLs can be classified as short, medium and long acyl chains based on the fatty acid chain lengths at *sn*-1 and *sn*-2 positions of the glycerol backbone. The molecular properties and biological reactivity of acyl chain are strongly influenced by the length of fatty acid chain [9]. Strong interested in understanding the mechanism of acyl group recognition by the enzyme, and further alteration of the acyl group specificity are rising. PLDs from *Streptomyces* species (PLDStr) are mostly used for this purpose due to their higher transphosphatidylation activity and broader substrate specificity compared to other microbial PLDs [10,11]. According to the NCBI database (https://www.ncbi.nlm.nih.gov/protein/?term=Streptomyces+Phospholipase+D) (accessed on 16 September 2021), currently there are 8099 protein sequences from *Streptomyces* in the PLD superfamily. However, until now, only about 20 PLDs from *Streptomyces* were experimentally characterized. *Streptomyces* are found in a wide range of aquatic and terrestrial environments, it have the metabolic pathways with the abundant hydrolytic enzymes [12,13]. However, most of the reported PLDs originated from *Streptomyces* live in terrestrial soil environments. Recently, the conventional screening and metagenome strategies have been developed to discover potential *Streptomyces* PLDs. Zhou et al. established 3 screening criteria for amino acid sequence identity, conserved motifs and availability of organism base on genome sequencing and annotation in the database. Based on 3 screening criteria, a new PLD was successfully mined from *Streptomyces mobaraensis*, which can catalyze soybean lecithin and L-serine into PS [14]. Although some achievements have been made, compared with a large amount of sequence information in this family, the extreme lack of enzymatic properties information serious hindered the in-depth understanding of the structure-function relationship and further application of this enzyme.

In the present study, a new PLD from marine *Streptomyces klenkii* (SkPLD) was biochemical characterized. With the help of molecular docking, the molecular basis for acyl chain specificity of SkPLD was illustrated. Meanwhile, the application potential of SkPLD for the synthesis of PS was evaluated. Present investigations not only further enriched the enzyme library but also provide important information on the understanding of the structure-function relationship of SkPLD.

## 2. Results

### 2.1. Bioinformatic Analysis of SkPLD

**Figure 1:**
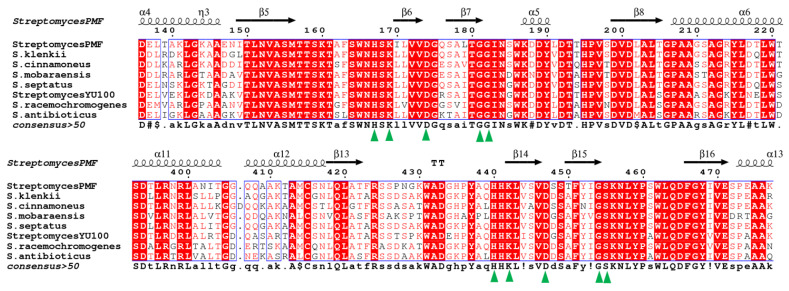
Multiple amino acid sequence alignment of SkPLD conserved region with several other *Streptomyces* PLDs. Secondary structures of PLDs were shown above the alignments. Residues highlighted in red background were indentical among the protein compared. Residues in the conserved HxKxxxxDxxxxxxGG/S (HKD) motifs of the PLD superfamily were indicated with green triangles. Arrow stands for β-strand, helical curve denotes α-helix.

The full-length SkPLD contains 539 amino acids and the first 33 amino acids were predicted to be the signal peptide (Figure S1), indicating that the enzyme was an extracellular protein. All known *Streptomyces* PLDs, except the *S. chromofuscus* PLD, contain two strictly conserved HKD motifs (HXK(X)_4_D(X)_6_GG/S), which are assumed to act as active center [15]. SkPLD showed substantially high identity to PLD protein sequences from *Streptomyces* (>70%) (Figure 1 and Figure S2). SkPLD also contained the typical active site of the PLD superfamily. Three catalytic residues (histidine, lysine and aspartic acid) of SkPLD formed the conserved sequence motif HXK(X)_4_D by the multiple alignment analysis.

### 2.2. Expression and Purification of SkPLD

The mature SkPLD (without signal peptides) was heterologously expressed in *E. coli* Shuffle T7 and then purified to homogeneity by using Ni^2+^ affinity chromatography, anion exchange chromatography and gel filtration chromatography (Figure S3). SDS-PAGE analysis revealed that recombinant SkPLD was approximately 54 kDa, which matched the theoretical molecular weight well. The protein contents and catalytic activities during the purification process were documented in Table S1. About 1.24 mg of pure SkPLD was obtained from 100 g wet cell weight, and the specific activity of pure SkPLD using soybean PC as substrate was determined to be 26.93 μmol/min/mg.

### 2.3. Enzymatic Characterization of SkPLD

**Figure 2:**
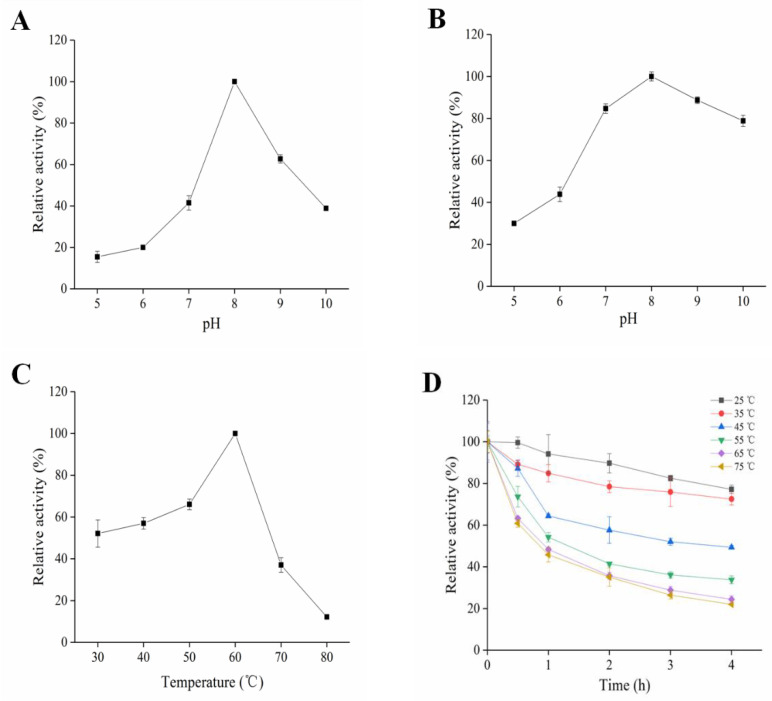
Effects of pH and temperature on the hydrolytic activity of SkPLD. (**A**) The enzymatic activity of SkPLD was measured under various pH buffers. The enzymatic activity value obtained at pH 8.0 was taken as 100%. (**B**) pH stability of SkPLD. The residual activity of SkPLD was determined after treating for 2 h under various pH buffers. The residual activity value obtained at pH 8.0 was taken as 100%. (**C**) The enzymatic activity of SkPLD was measured at various temperatures. The enzymatic activity value obtained at 60 °C was taken as 100%. (**D**) Thermostability of SkPLD. The residual activity was determined after treating for 4 h at different temperatures. Values were means ± standard deviation from three independent experiments.

SkPLD was found to be an alkaline PLD, with a maximal activity at pH 8.0, and exhibited lower (about 40% of the maximum activity) at pH 7.0 and pH 10.0 (Figure 2A). SkPLD showed wide pH stability between 7.0–10.0, with more than 75% of residual activities left after 2 h treatment under corresponding pH buffers (Figure 2B). As shown in Figure 2C, the maximum hydrolytic activity of SkPLD was found at 60 °C, whereas its activity decreased to 40% of the maximum under the temperature of 70 °C. SkPLD kept stable after incubation at temperatures lower than 35 °C for 4 h, with a residual activity higher than 80% (Figure 2D). SkPLD kept more than 84.75% of the initial activity while the existence of different metal ions (10 mM) in the buffer, including Na^+^, K^+^, Cu2^+^, Li^+^ and Zn^2+^. As metal ions concentration increases from 1 mM to 5 mM, the hydrolytic activity of SkPLD was activated in the presence of Co^2+^, Ca^2+^ and Mn^2+^ but was inhibited by Al^3+^ and Fe^2+^. The enzyme activity of SkPLD kept 85.66% even in the existence of 10 mM EDTA (Table S2).

### 2.4. Kinetic Parameters and Acyl Chain Specificity of SkPLD

The kinetic parameters of SkPLD were determined with soybean PC or PCs with different chain lengths as substrate. The enzyme showed hyperbolic *v*/[*S*]-curves, which could be fitted well according to the classical Michaelis-Menten function by non-linear regression (Figure S4). The apparent *V_max_* and *K_m_* values were determined to be 41.65 ± 1.84 μmol/min/mg and 1.33 ± 0.13 mM, respectively. The influence of the acyl chain length in PC on the kinetic parameters of SkPLD was also investigated (Table 1). The enzymes exhibited the highest catalytic efficiency (*k*_cat_/*K*_m_) toward PCs with medium acyl chain followed by short and long acyl chain. SkPLD showed a maximum catalytic efficiency (*k*_cat_/*K*_m_) toward the 12:0/12:0-PC (67.13 S^−1^ mM^−1^) and *k*_cat_/*K*_m_ value decreased sharply as the substrate changed from 12:0/12:0-PC to 18:0/18:0-PC.

### 2.5. Structural Feature and Molecular Basis for Chain Length Specificity

**Figure 3:**
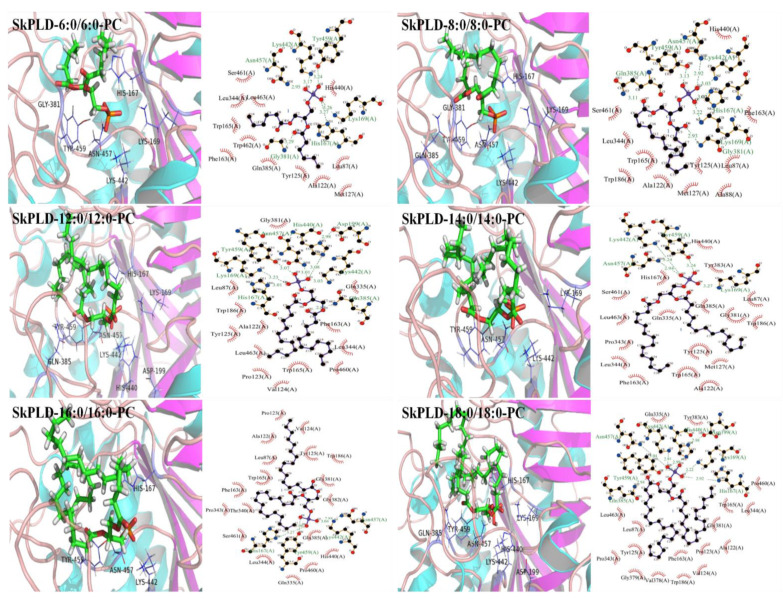
3D (**left**) and 2D (**right**) structure of complexes of SkPLD and 6:0/6:0-PC, 8:0/8:0-PC, 12:0/12:0-PC, 14:0/14:0-PC, 16:0/16:0-PC and 18:0/18:0-PC. In the 2D diagram, hydrogen-bond interactions and hydrophobic contacts were shown as green and red dotted lines, respectively.

The structural quality of the model for SkPLD was validated to ensure that it was suitable for performing further studies. As shown in Figure S5A, the overall quality of the model was also evaluated by the score obtained from QMEAN server. QMEAN4 score, which is a composite score consisting of a linear combination of 4 statistical potential terms. This value falls in the range of estimated model reliability score between 0 and 1. The normalized QMEAN4 Z-score for the model was found to be 0.87. Thus, the quality evaluation using online tools QMEAN confirmed a proper model conformation and of good quality for further analysis. As shown in Figure S5B, SkPLD contained the catalytic quadruplex amino acid residues His167, Lys169, Asn184, His440, Lys442 and Asn457. They were in similar locations to those of the crystal structure of PLD from *S. antibioticus* (PDB ID: 2ZE4) and *Streptomyces* sp. PMF (PDBID: 1F0I), demonstrating a proper model conformation. The docking study of the substrate PCs against PLD was performed, and the results are displayed in Figure 3 and Table 2. The hydrogen-bond interactions between SkPLD and PCs was initially increased with increasing length of acyl chain from 6:0/6:0-PC to 12:0/12:0-PC and then decreased with increasing length of acyl chain from 12:0/12:0-PC to 16:0/16:0-PC. The absolute value of estimated binding energy increased from 7.10 to 7.54 kcal/mol and then decreased from 7.54 to 6.79 kcal/mol for the SkPLD-PCs complex as increasing chain length from 6:0/6:0-PC to 16:0/16:0-PC. SkPLD and 16:0/16:0-PC complex showed four hydrogen bonds. However, SkPLD and 12:0/12:0-PC complex had eight hydrogen bond with an estimated binding energy of −7.54 kcal/mol, which is the highest binding affinity. SkPLD and 18:0/18:0-PC complex obtained binding energy of −7.28 kcal/mol and relatively high hydrogen-bond interaction, which were consistent with its substrate affinity in Table 1.

### 2.6. Enzymatic Synthesis of PS by Recombinant SkPLD

**Figure 4:**
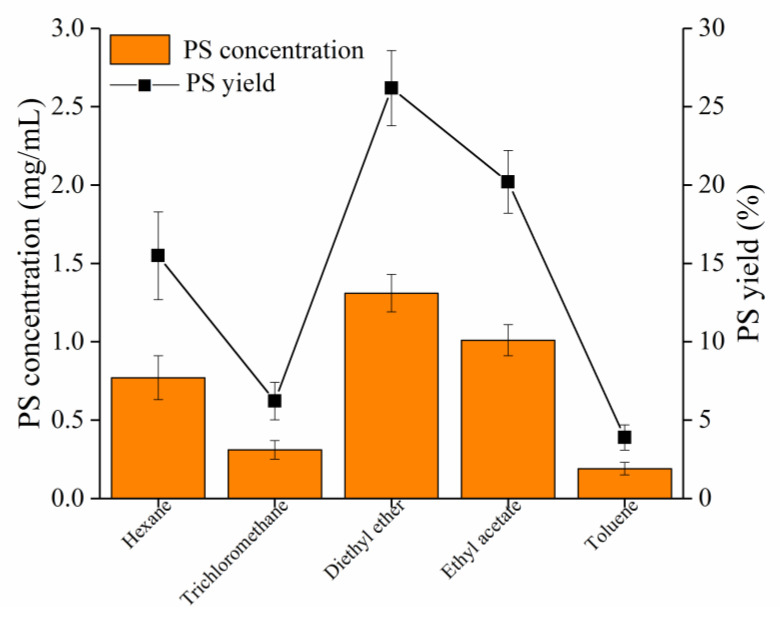
Application of SkPLD for PS synthesis. Effect of different organic solvents on the yield of PS. Values were means ± standard deviation from three independent experiments.

PLD-catalyzed synthesis of PS from soybean PC is a transphosphatidylation reaction in which PC is a donor of the phosphatidyl residue and L-serine is an acceptor. PS could be synthesized with L-serine and soybean PC as substrates under the catalysis of SkPLD. Effects of the type of organic solvent were investigated to optimize the PS yield. As shown in Figure S6, the addition of SkPLD would generate the desired product PS, as well as undesired by-product PA. As shown in Figure 4, using diethyl ether as the organic phase, with shaking (200 rpm) at 40 °C for 12 h, the reaction showed 26.18% of yield and 1.31 mg/mL of accumulated PS concentration. The reaction in the diethyl ether afforded 1.70, 4.23, 1.30 and 6.89 times PS yield as much as that of the hexane, tricholoromethane, ethyl acetate and toluene.

## 3. Discussion

### 3.1. Bioinformatic Analysis of SkPLD

General findings related to the PLD catalytic reactions are a “ping-pong” type of reaction by the two HKD motifs. The histidine residue of the HKD motif in one subunit acts as the nucleophile attacking the phosphate of the phosphodiester bond, and the histidine residue in the other HKD motif acts as a general acid protonating the leaving group [16]. GG/S sequence was identified at the seven amino acids downstream of 167HXK(X)_4_D174 and 440HXK(X)_4_D447 motif from SkPLD sequence. Two GG/S motifs patterns have also been found in other PLD sequences from *Streptomyces*. The PLDs from *Streptomyces* was known for higher catalytic activity, which was likely to benefit from the function of two GG/S motifs. Ogino et al. investigated that the G215S and G216S mutants exhibit approximately 9–16 folds higher transphosphatidylation activity than the wild type PLD. However, the S489G mutant show reduced activity and G488S lost the activity completely [17]. In another study, it was inferred from the crystal structure of *Streptomyces* sp. Strain PMF PLD that the GG/GS motif might control the active centre cleft’s conformation or access [18]. These findings implied that SkPLD carrying the GG/S motifs might also recognize phospholipids more widely and affect the activity of the transphosphatidylation reaction.

### 3.2. Enzymatic Characterization of SkPLD

SkPLD showed suitable hydrolyzing activity in the pH range between 6.5 and 8.5, which was similar to other reported microbial PLDs from *Streptomyces* [19]. SkPLD showed wide pH stability between 7.0–10.0. However, the PLD of *Streptomyces* sp. PMF and *Streptomyces* sp. CS-57 undergo rapidly decreased activity when pH value was higher than 9.0 [20,21]. The maximum hydrolytic activity of SkPLD was found at 60 °C, whereas its activity decreased to 40% of the maximum under the temperature of 70 °C. SkPLD kept stable after incubation at temperatures lower than 35 °C for 4 h, with a residual activity higher than 80%. The PLD of *Streptomyces* sp. PMF was stable between 20 °C and 50 °C. However, while treated at 60 °C for 3 h, its residual activity was significantly lost [18]. The enzyme from *S. halstedii* K1 lost its activity at 45 °C. PLD from *S. septatus* TH-2 was stable between 15 °C and 60 °C, but decrease rapidly at 70 °C [22]. Compared to the above mentioned PLDs, SkPLD showed higher thermostabilty, especially at temperatures higher than 60 °C.

### 3.3. Kinetic Parameters and Acyl Chain Specificity of SkPLD

Knowledge of substrate specificity is essential to improve the quality of the products and to further elucidate the mechanisms of reaction. The influence of the acyl chain length in PC on the kinetic parameters of SkPLD was also investigated (Table 1). The enzymes exhibited the highest catalytic efficiency (*k*_cat_/*K*_m_) toward PCs with medium acyl chain followed by short and long acyl chain. This may be due to the increased steric hindrance of long acyl-chains in the substrate-binding pockets and differences in hydrophobic interaction between the acyl chains and substrate-binding pockets [23,24]. Until now, the effect of acyl chain length of PC substrates on the *S. antibioticus* PLD have been studied [25]. *S. antibioticus* PLD shows relatively high specific activity toward PCs with medium and long *sn*-2 acyl chain, and remarkably decreases toward PCs short *sn*-2 acyl chain. In summary, PCs with short and medium acyl chain were better substrates for SkPLD than those with longer chains, this characterization may be enable synergies between SkPLD and SaPLD in industrial applications that require a wider substrate range.

### 3.4. Structural Feature and Molecular Basis for Chain Length Specificity

In order to explore the molecular basis of SkPLD-mediated chain length specificity, the docking study of the substrate PC against PLD was performed, and the results are displayed in Figure 3 and Table 2. Hydrogen bond is one of the most important directional intermolecular interactions, it is responsible for the basic structures and stabilities of biomolecules [26]. Although the geometry of catalytic site is highly conserved, the binding pockets are highly variable among enzymes. The variable composition of the binding pockets generates an elastic environment for the acyl chains with various lengths [27]. The hydrogen bonds between SkPLD and PCs were initially increased with increasing length of acyl chain from 6:0/6:0-PC to 12:0/12:0-PC and then decreased with increasing length of acyl chain from 12:0/12:0-PC to 16:0/16:0-PC, which were basically consistent with its catalytic efficiency (*k*_cat_/*K*_m_) and substrate affinity (*K*_m_). This can be explained that PCs with medium chain lengths provided more binding sites to the protein chain, causing stronger hydrogen bond interactions. Generally, the hydrophobicity of the bottom of the substrate-binding pocket is important for both selectivity and recognition of hydrophobic substrates [28]. The hydrophilic regions of substrate-binding pocket of SkPLD were mainly located in the amino acid sequences of 381–385. Compared with PCs with short and medium acyl chain lengths, the hydrophobic acyl chain of 16:0/16:0-PC was in contacted with the hydrophilic regions (Gly381, Gly382 and Gln385) of substrate-binding pocket of SkPLD. These contacts may weaken hydrophobic interactions and which are detrimental to hydrogen bond formation and complex stability [29]. SkPLD and 18:0/18:0-PC complex obtained binding energy of −7.28 kcal/mol and relatively high hydrogen-bond interaction, which was consistent with its substrate affinity (*K*_m_). However, SkPLD exhibited low catalytic efficiency for 18:0/18:0-PC. This can be explained that the increased steric hindrance of long acyl-chains in the substrate-binding pockets and it was more difficult for PC molecule to enter and escape the binding pocket. These results indicated that PCs with shorter chain length provided less binding sites to the protein chain, causing weaker interactions or binding, while the PCs with long chain length increased steric hindrance in the substrate-binding pocket.

### 3.5. Enzymatic Synthesis of PS by Recombinant SkPLD

Since both the substrate PC and the product PS are water-insoluble while the substrate L-serine is water-soluble, this enzyme-catalyzed transphosphatidylation generally was carried out in a biphasic system consisting of a water-immiscible organic solvent phase [30]. Plenty of organic solvents have been well investigated for the *Streptomyce* PLD-mediated transphosphatidylation for PS preparation, including diethyl ether, ethyl acetate, chloroform and dichloromethane. *S. racemochromogenes* PLD used dichloromethane as preferred organic solvent phase [31]. *Streptomyces* sp. SC734 PLD and *Streptomyces chromofuscus* AS 4.331 used chloroform as preferred organic solvent phase [32,33]. *Streptomyces* sp. CA-1 and *Streptomyces* sp. used ethyl acetate as preferred organic solvent phase [34,35]. Notably, most of *Streptomyces* PLD have achieved more than 50% of PS using diethyl ether as organic solvent phase, such as *Streptomyces* sp. YU100, *Streptomyces prunicolor*, *Streptomyces* sp. LD0501 [36,37]. Although diethyl ether system have achieved decent yields of PS, the enzymatic reaction is greatly affected by plenty of factors, such as the choice of organic solvent, the volume ratio of biphasic system, the proportion of substrate concentration, pH, temperature, which make it much complicated [38]. Moreover, diethyl ether has certain toxicities which lead to enzyme denaturation [39]. Therefore, further optimization of *Streptomyces* PLD-catalyzed transphosphatidylation condition, including culture time, the volume ratio of biphasic system, pH and temperature are necessary for industrial applications in PS production.

## 4. Materials and Methods

### 4.1. Chemicals, Strains, and Materials

PC (purity 95%, from soybean), 1, 2-dihexanoyl-*sn*-glycero-3-phosphocholine (6:0/6:0-PC) (purity > 99%), 1, 2-dioctanoyl-*sn*-glycero-3-phosphocholine (8:0/8:0-PC) (purity > 99%), 1, 2-dilauroyl-*sn*-glycero-3-phosphocholine (12:0/12:0-PC) (purity > 99%), 1, 2-dimyristoyl-*sn*-glycero-3-phosphocholine (14:0/14:0-PC) (purity > 99%), 1, 2-di-palmitoyl-*sn*-glycero-3-phosphocholine (16:0/16:0-PC) (purity > 99%) and 1, 2-distearoyl-*sn*-glycerol-3-phosphocholine (18:0/18:0-PC) (purity > 99%) were purchased from Avanti Polar Lipids, Inc. (Alabaster, AL, USA). PS standard (purity > 97%) was purchased from Sigma-Aldrich (St. Louis, MO, USA). Choline oxidase was prepared by the previously reported method [40]. Horseradish peroxidase was purchased from Sangon Biotech Co., Ltd. (Shanghai, China). Hypersil GOLD Silica column (Dim. 4.6 × 250 mm, particle size 5.0 µm) was obtained from Thermo Fisher Scientific (Waltham, MA, USA). *Escherichia coli* SHuffle T7 Express competent cell were purchased from New England BioLabs (Beijing, China). The plasmid pET28a vector was purchased from Invitrogen (Carlsbad, CA, USA). Ni^2+^-nitrilotriacetate (Ni^2+^-NTA) affinity column, desalting column, Q-Sepharose column and Hiload 16/60 Superdex 200 pg gel filtration column were obtained from GE Healthcare Life Sciences (Pittsburgh, PA, USA). All other chemicals used in the present study were analytical grade.

### 4.2. Bioinformatic and Homology Modeling

The SkPLD protein from marine *Streptomyces klenkii* that expressed in this study was deposited in the NCBI-Protein databases under the accession number of RKN69773.1. The signal peptide of SkPLD was predicted using online tools SignaIP v. 5.0 web server (http://www.cbs.dtu.dk/services/SignalP/, accessed on 20 March 2020) [41]. The mature peptide (34 to 539aa, without signal peptide) of SkPLD protein was codon optimization with the *E. coli* code usage (Figure S7). Sequence comparison of SkPLD to other known PLDs was performed by Multalin (http://multalin.toulouse.inra.fr/multalin/, accessed on 20 March 2020) [42]. Homology modeling of SkPLD was performed using the SWISS-MODEL (https://swissmodel.expasy.org, accessed on 20 March 2020) online system [43]. The protein three-dimensional structure of PDB entry 2ZE9 with the sequence identity of 75.56% to SkPLD was used as a template [44]. The validation and quality estimation of predicted SkPLD model was evaluated by QMEAN [45]. The ligand and protein files were prepared using AutoDock tools [46]. AutoDock tools was used for protein-ligand docking and the resulting interactions of between receptor and ligand were visualized PyMOL (version 2.5) [47] and LigPlus (version 2.2) [48].

### 4.3. Recombinant Expression of SkPLD in Escherichia coli

The gene encoding the mature protein of SkPLD without the signal peptide was artificially synthesized by Sangon Biotech Co., Ltd. (Shanghai, China) and inserted into the pET-28a expression vector. Plasmids were confirmed by sequencing. The constructed vector was further transformed into Shuffle T7 express competent *E. coli*. A single colony of *E. coli* SHuffle T7 cells harbouring the plasmid pET-28a-SkPLD was grown at 37 °C for 12 h in 5 mL of Luria-Bertani medium containing 50 μg/mL kanamycin. An aliquot (5 mL) of this culture was inoculated into 500 mL of the same medium and grown at 37 °C with shaking (220 rpm). When the OD_600_ value reached 0.6 (Metash UV-6000PC, Shanghai, China), enzyme expression was induced by adding isopropyl-β-D-thiogalactopyranoside (IPTG) with a final concentration of 1 mM. After cultivation was continued for another 12 h at 16 °C, cells were harvested by centrifugation (Hermle Z326K, Wehingen, Germany) at 8000× *g* for 10 min at 4 °C and stored at −20 °C for further use.

### 4.4. Purification of Recombinant SkPLD

The SkPLD was purified by metal-chelating chromatography using a Ni^2+^-nitrilotriacetate affinity column. Buffer A (50 mM Tris-HCl, 20 mM imidazole, pH 8.0) containing crude enzyme was loaded into a Ni^2+^-nitrilotriacetate affinity column and washed with buffer B (50 mM Tris-HCl, 250 mM imidazole, pH 8.0). The eluant was buffer exchanged to 20 mM Tris-HCl (pH 8.0) through a desalting column. And then applied to a Q-column pre-equilibrated with 20 mM Tris-HCl, pH 8.0. The elution was performed at the flow rate of 1 mL/min with a gradient of NaCl from 0 to 1 M, in 20 mM Tris-HCl, pH 8.0. Then we knowed that buffer containing 700 mM NaCl can elude the target protein. Therefore, we eluted the non-target protein with buffer containing 500 mM NaCl, and then eluted the target protein with buffer containing 700 mM NaCl to obtain a relatively pure target protein. The eluted sample from Q-column chromatography with elution buffer containing 700 mM NaCl was buffer exchanged to 20 mM Tris-HCl (pH 8.0) through a desalting column and then applied to a Hiload 16/60 Superdex 200 pg. Finally, target protein fractions were further pooled after size-exclusion chromatography. The absorbance of the elution was monitored at 280 nm.

### 4.5. Enzyme Activity Assay

The PLD activity was assayed using the method described by Shimbo et al. with minor modifications [49]. For substrate preparations, 1 mmol of soybean PC or corresponding amounts of other phospholipids were dissolved in 5 mL of chloroform. After evaporation to dryness, SDS and Triton X-100 were added to yield a solution containing 10 mM PC, 12.5 mM SDS, and 40 mM Triton X-100. The solution was vortexed and sonicated for 10 min using a probe sonicator (800 W/s). The reaction mixture (100 μL) consisted of 4 mM soybean PC, 50 mM Tris-HCl (pH 8.0), 20 mM CaCl_2_ and 10 μL of an enzyme sample. After incubation at 40 °C for 10 min with shaking, reaction was terminated by adding 25 μL Tris-HCl (50 mM, pH 8.0) solution that contained 50 mM EDTA. The PLD was further denatured by heating at 100 °C for 5 min. After cooling the reaction mixture to room temperature for 5 min, 25 μL of Tris-HCl (50 mM, pH 8.0) containing 42 mM phenol, 50 mM 4-aminoantipyrine, 0.5 U of horseradish peroxidase and 0.25 U of choline oxidase was added. After incubation at 37 °C for 60 min, the absorbance of the reaction mixture was measured at 500 nm (Tecan Infinite 200 PRO, Männedorf, Switzerland). As a blank test, 10 μL of distilled water was used instead of the enzyme solution, and the reaction mixture was treated in the same way. The calibration curve was obtained using a standard solution of choline chloride instead of the enzyme solution. One unit (U) of hydrolytic activity of PLD was defined as the amount of enzyme producing 1 μmol of choline per minute under these assay conditions. All assays were performed in triplicate.

### 4.6. Enzymatic Characterization of SkPLD

Optimum pH for the purified SkPLD activity was determined at 40 °C using soybean PC as substrate. pH buffer solutions ranged from 5.0 to 10.0 (pH 5.0, 0.1 M sodium citrate-citric acid; pH 6.0–7.0, 0.1 M phosphate buffer; pH 8.0, 0.1 M Tris-HCl and pH 9.0–10.0, 0.1 M Gly-NaOH). pH stability of SkPLD was determined by pre-incubating enzymes in different pH buffers for 2 h at 25 °C, and then the residual activity was determined at 40 °C and optimum pH. The optimum temperature of the SkPLD activity was determined at optimum pH. The temperature range was set from 30–80 °C. The thermostability of SkPLD was determined by pre-incubating the enzyme at different temperatures and pH 8.0, and then samples were taken every 60 min for measurement of residual activity under the above assay optimum conditions. The temperature was set as 25, 35, 45, 55, 65 and 75 °C, respectively. Various metal ions (Mg^2+^, Co^2+^, Ca^2+^, Na^+^, Mn^2+^, Al^3+^, K^+^, Cu^2+^, Li^+^, Zn^2+^, Fe^2+^ and Fe^3+^) and EDTA at final concentrations of 10 mM were added to the enzyme at pH 8.0, and then the PLD activity was assayed after pre-incubation at 25 °C for 2 h. The enzyme activity in the absence of metal ions and EDTA were defined as 100%. 

The kinetic parameters of SkPLD to soybean PC and PC with different defined fatty acids (6:0/6:0, 8:0/8:0, 12:0/12:0, 14:0/14:0, 16:0/16:0, 18:0/18:0) as the substrates were determined by using enzyme-linked colorimetry with different substrate concentrations (0–4 mM). The affinity constant (*K*_m_), maximal velocity (*V*_max_), and turnover number (*k*_cat_) values of the reaction were obtained using the Michaelis-Menten equation. The independently replicated determination was performed three times for each substrate. The error bars represent three independent assays of kinetic parameters.

### 4.7. Application of SkPLD for the PS Synthesis

Reactions were performed in a biphasic reaction system. The initial conversion conditions were as follows: a reaction mixture consisting of 20 mg/mL L-serine in 500 μL enzyme solution (100 μg/mL PLD in 100 mM Tris-HCl buffer, pH 8), and soybean PC (5 mg/mL) dissolved in 500 μL of organic solvent was incubated at 40 °C for 12 h with shaking. To obtain the optimum PLD-catalyzed transphosphatidylation conditions, the effects of various organic solvent (hexane, ethyl acetate, chloroform, ether and toluene) on the yield of PS were investigated. For quantification, PS was detected by Waters 1525 HPLC (Milford, MA, USA) equipped with ELSD. Reaction mixture was eluted with a column using solvent A (methanol/water/acetic acid/triethylamine, 425:75:2.5:0.25, by vol.) and solvent B (n-hexane/isopropanol/solvent A, 160:384:256, by vol.). The elution profile was as follows: 0 min, A = 100%; 9 min, B increased to 40%; 13 min, B increased to 60%; 17 min, B = 100%; 22 min decreased to 0%. The yield of PS (%) was defined as the percentage of PS obtained compared with the initial PC concentration.

## 5. Conclusions

In summary, a new PLD from marine *Streptomyces klenkii* was biochemical characterized. PCs with short and medium acyl chain were better substrates for SkPLD than those with longer chains. The different catalytic efficiency of SkPLD toward PCs with various chain lengths was related to the increased steric hindrance of long acyl-chains in the substrate-binding pockets and differences in hydrogen-bond interactions between the acyl chains and substrate-binding pockets. Present investigations not only further enriched the enzyme library but also provide guidance for the further mining of PLDs with special phospholipids recognition properties.

## Figures and Tables

**Table 1:** Kinetic parameters for the hydrolysis of SkPLD towards different substrates.

Substrates	*K*_m_ (mM) ^a^	*k*_cat_ (S^−1^) ^b^	*k*_cat_/*K*_m_ (S^−1^ mM^−1^) ^c^
6:0/6:0-PC	2.01 ± 0.18	100.85 ± 4.42	50.27
8:0/8:0-PC	2.59 ± 0.28	110.54 ± 6.30	42.63
12:0/12:0-PC	1.09 ± 0.10	73.29 ± 2.68	67.13
14:0/14:0-PC	1.07 ± 0.18	61.65 ± 4.05	57.51
16:0/16:0-PC	2.01 ± 0.19	32.97 ± 1.48	16.43
18:0/18:0-PC	1.27 ± 0.14	15.45 ± 0.69	12.12

^a^ *K*_m_: the substrate affinity constant. ^b^ *k*_cat_: the turnover of the enzymatic reaction. ^c^ *k*_cat_/*K*_m_: the catalytic efficiency.

**Table 2:** The estimated binding energy of SkPLD-PC complexes.

Complex	Binding Energy (kcal/mol)
SkPLD-6:0/6:0-PC	–7.10
SkPLD-8:0/8:0-PC	−7.13
SkPLD-12:0/12:0-PC	−7.54
SkPLD-14:0/14:0-PC	−7.29
SkPLD-16:0/16:0-PC	−6.79
SkPLD-18:0/18:0-PC	−7.28

## Data Availability

The data presented in this study are available on request from the corresponding author.

## Supplementary Materials

The following are available online at https://www.mdpi.com/article/10.3390/ijms221910580/s1.
