# Supplementary material for: Acyl Chain Specificity of Marine Streptomyces klenkii PhosPholipase D and Its Application in Enzymatic Preparation of Phosphatidylserine
Source: Int J Mol Sci. 2021 Sep 30;22(19):10580. doi: 10.3390/ijms221910580 (PMC8508628; doi:10.3390/ijms221910580)
Supplement: Supplementary file 1 [file ijms-22-10580-s001.zip › ijms-1367105-supplementary.pdf]

Supplementary material

Acyl chain specificity of marine *Streptomyces klenkii* phospholipase D and its application in enzymatic preparation of phosphatidylserine

Rongkang Hu, Ruiguo Cui, Dongming Lan, Fanghua Wang*, Yonghua Wang*

School of Food Science and Engineering, South China University of Technology, Guangzhou, Guangdong 510640, People's Republic of China

*To whom correspondence should be addressed: Yonghua Wang, yonghw@scut.edu.cn; Fanghua Wang, wangfanghua@scut.edu.cn.

Table S1. Purification table of SkPLD.

Fraction	Total activity (µmol/min)	Total protein (mg)	Specific activity (µmol/min/mg)	Purification fold	Yield (%)
Lysate	104.91	654.08	0.16	1	100
Ni-column chromatography	72.75	31.93	2.28	12.5	69.35
Q-column chromatography	34.72	7.59	4.57	25	33.1
Hiload 16/60 Superde 200 pg	33.48	1.24	26.93	50	31.91

Table S2. Effects of various metal ions and EDTA on the hydrolytic activity of SkPLD.

Metal ions	Relative activity (%)（5mM）	Relative activity (%)（10mM）
Control	100 ± 2	100 ± 1
Co^{2+}	112 ± 4	124 ± 9
Ca^{2+}	113 ± 4	117 ± 2
Mn^{2+}	107 ± 1	115 ± 1
Na^{+}	102 ± 4	101 ± 4
Li^{+}	90 ± 5	90 ± 11
K^{+}	94 ± 1	87 ± 3
Zn^{2+}	96 ± 1	87 ± 4
Cu^{2+}	92 ± 3	85 ± 4
Mg^{2+}	94 ± 4	79 ± 2
Fe^{3+}	78 ± 2	70 ± 7
Fe^{2+}	80 ± 2	43 ± 6
Al^{3+}	82 ± 2	43 ± 1
EDTA	90 ± 1	86 ± 2

Figure S1. The signal peptide prediction of SkPLD.

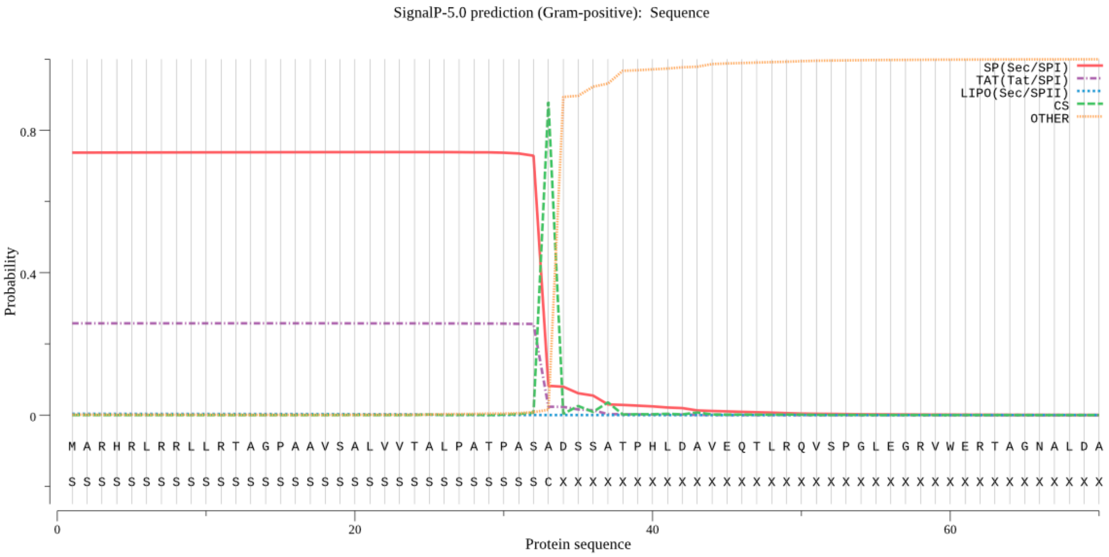

Figure S2. Multiple amino acid sequence alignment of SkPLD with several other *Streptomyces* PLDs. Secondary structures of PLDs were shown above the alignments. Residues highlighted in red background were indentical among the protein compared. Residues in the conserved HxKxxxxDxxxxxxGG/S (HKD) motifs of the PLD superfamily were indicated with green triangles.

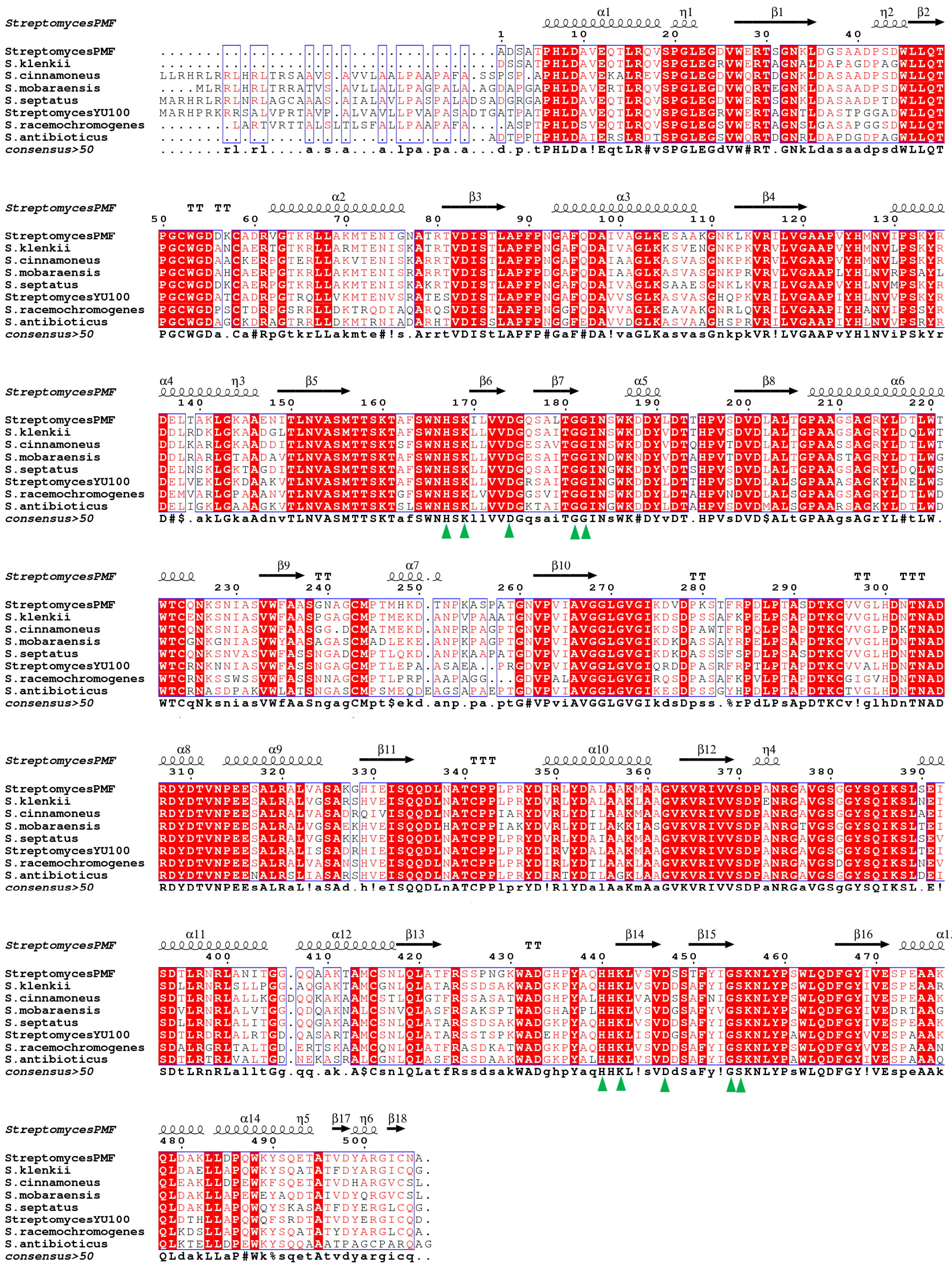

Figure S3. (A) SDS-PAGE analysis of the recombinant SkPLD overexpressed in *Escherichia coli* SHuffle T7. Lane M, protein molecular weight marker. Lane 1, crude enzyme. Lane 2, the sample was eluted directly from Ni^{2+} affinity chromatography with elution buffer containing 250 mM imidazole. Lane 3, the sample was eluted directly from Q-column chromatography with elution buffer containing 700 mM NaCl. Lane 4, pooled target proteins from Hiload 16/60 Superdex 200 pg gel filtration column. (B) The size-exclusion chromatogram for Hiload 16/60 Superdex 200 pg.

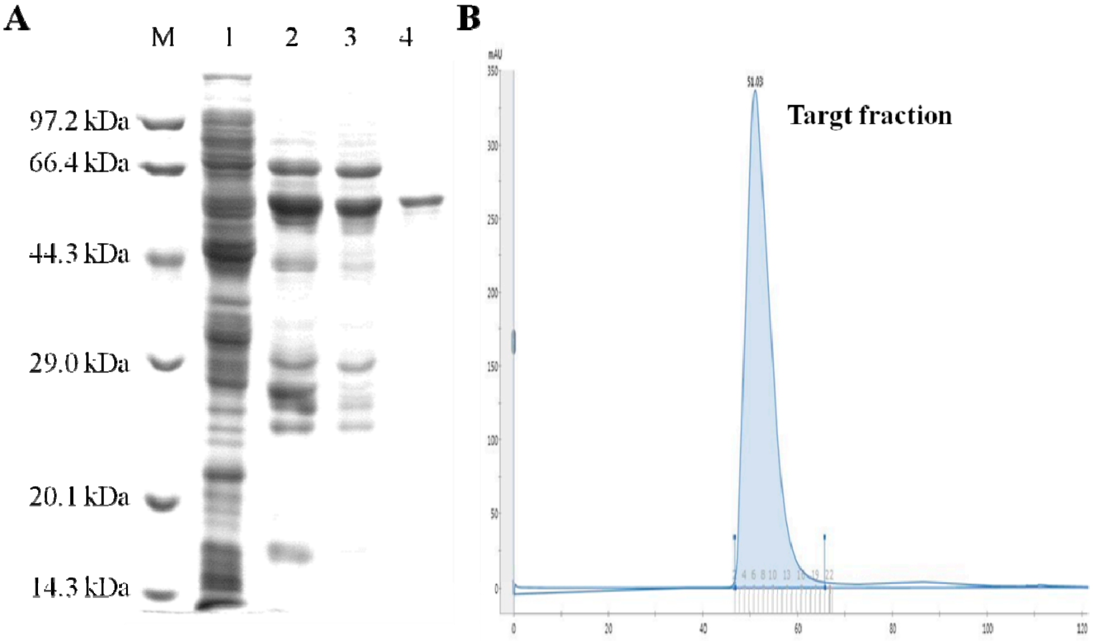

Figure S4. PLD activity as a function of substrate concentration. *v*/[*S*] characteristics of the PLD were determined in a mixed micellar system.

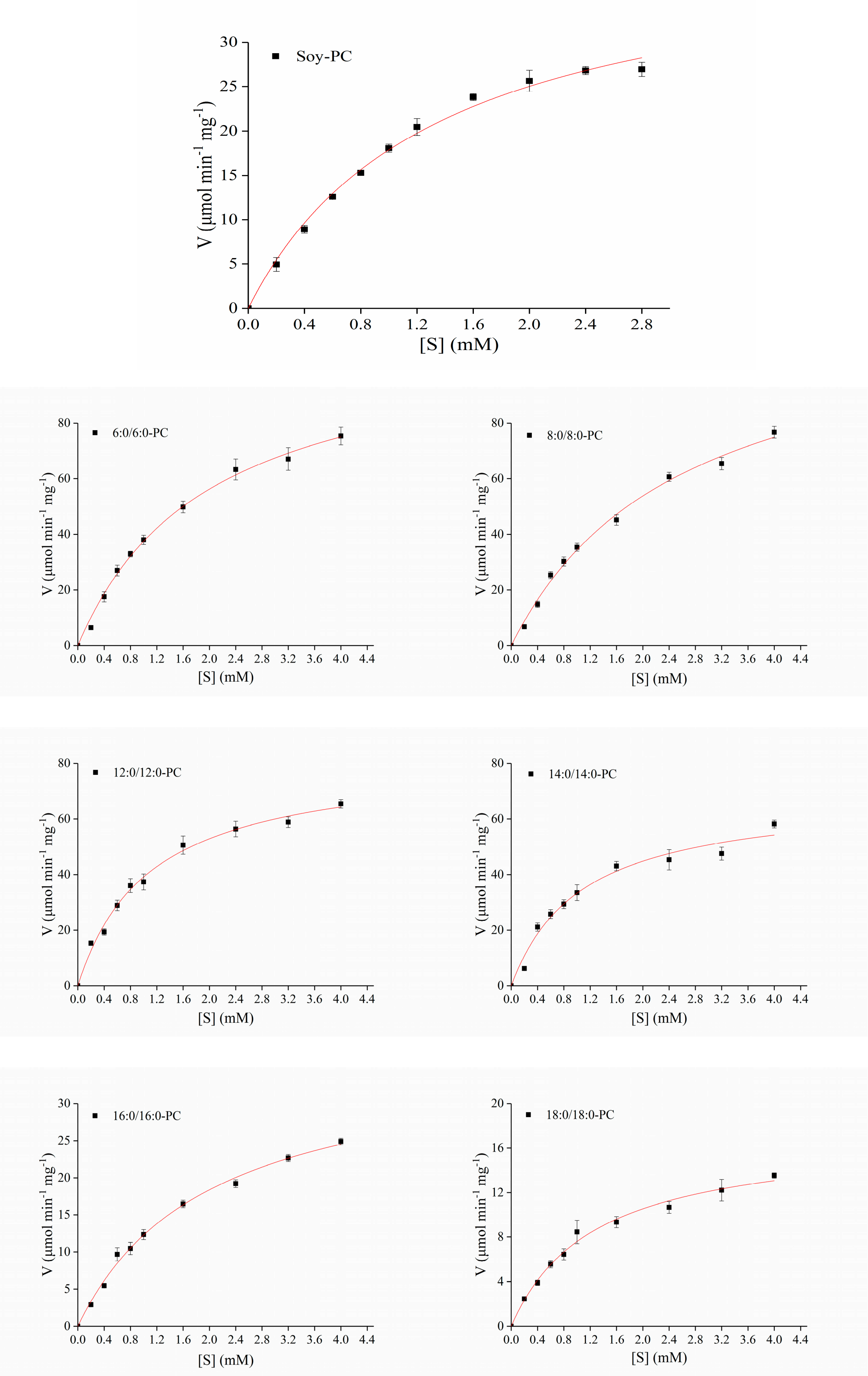

Figure S5. Structural verifcation homology modeling model of the SkPLD. (A) The results of structural verifcation of SkPLD by QMEAN. (B) The catalytic quadruplex amino acid residues were found in SkPLD (green) and showed high structural consistence with *Streptomyces sp.* PMF PLD (magentas, PDB ID: 1F0I) and *S. antibioticus* PLD (cyan, PDB ID: 2ZE4).

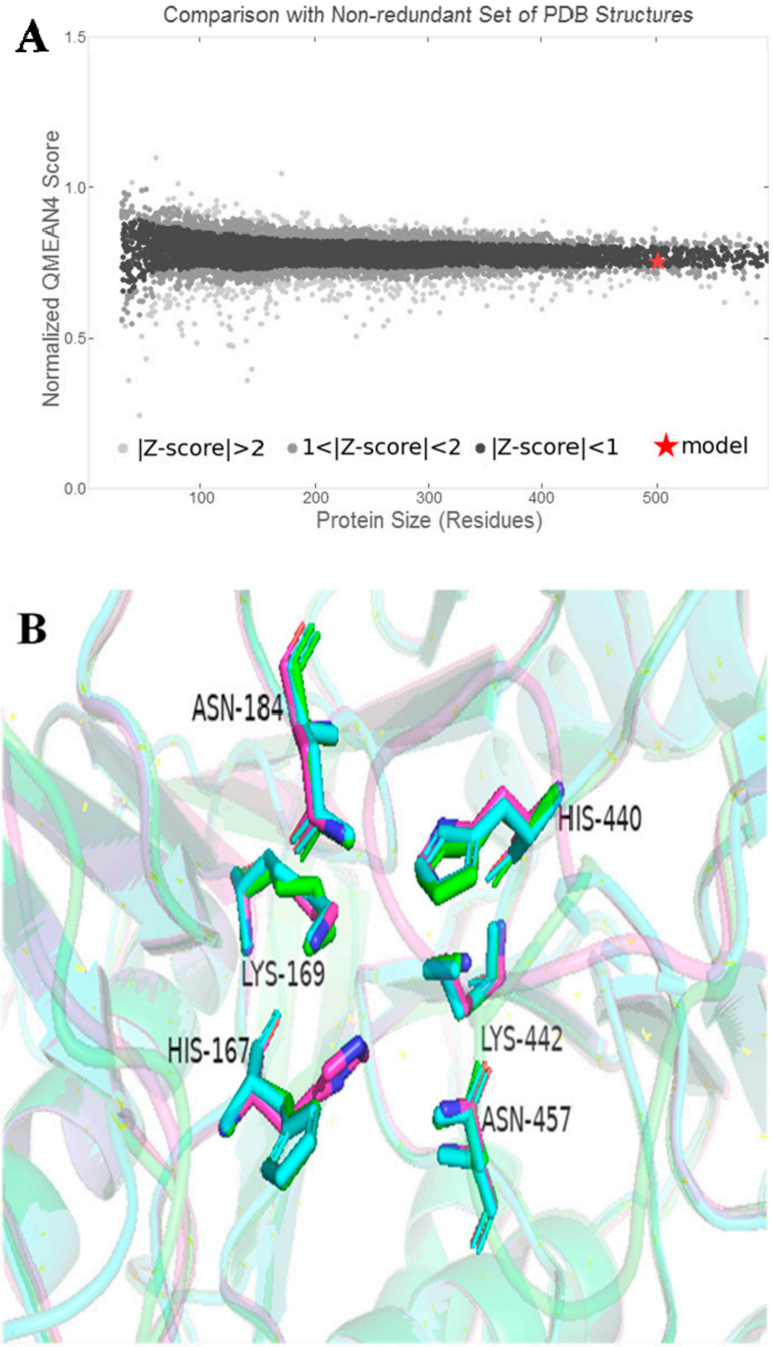

Figure S6. Determination of phosphatidylserine by HPLC. A: The blank sample subjected to HPLC using ELSD. B: The phosphatidylserine standard sample. C: The phosphatidic acid standard sample; D: The phosphatidylcholine standard sample; E: The reaction mixtures were subjected to HPLC using ELSD for phosphatidylserine determination.

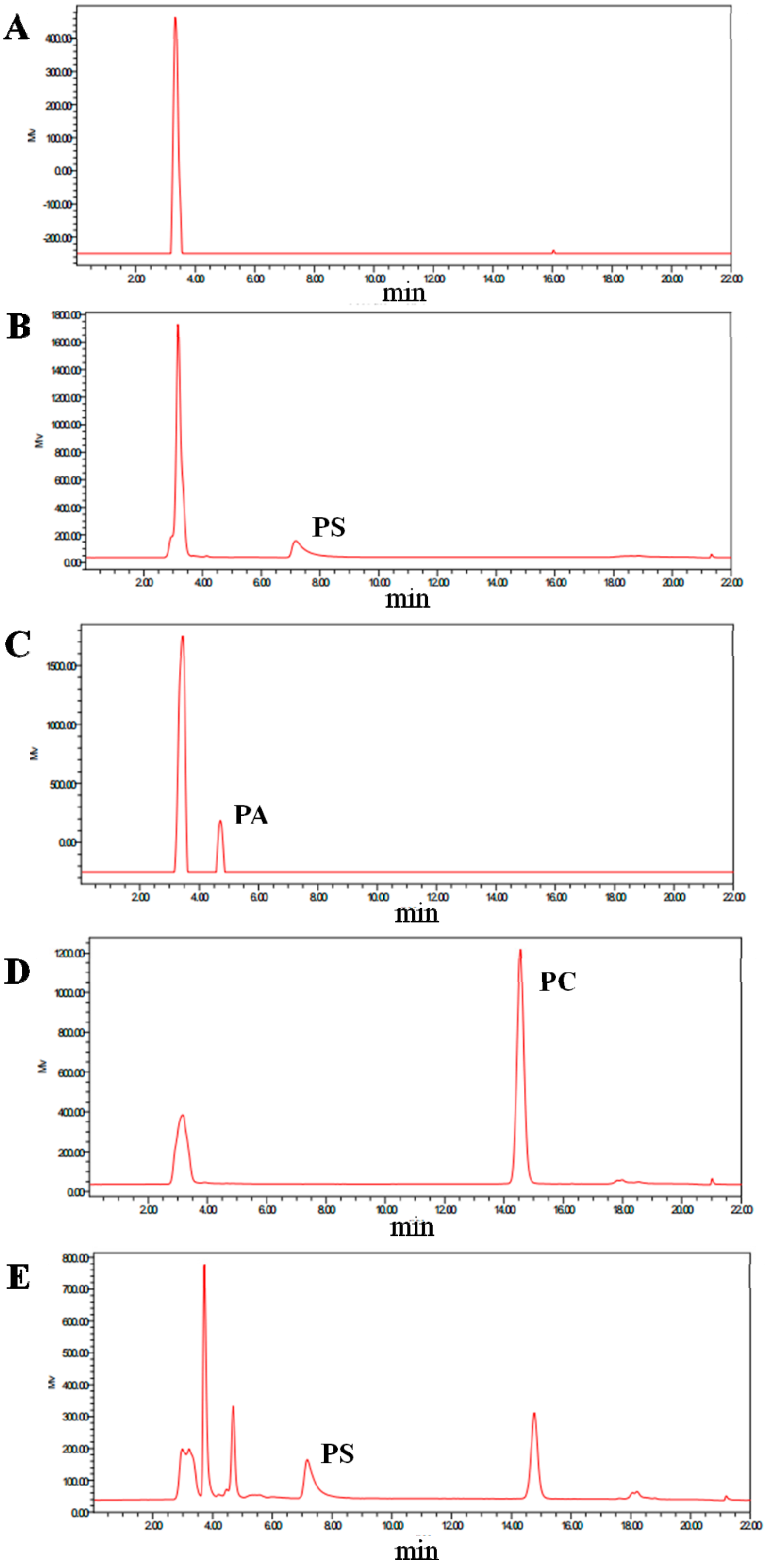

Figure S7. Codon optimization the mature peptide (34 to 539aa, without signal peptide) of SkPLD protein with the *E. coli* code usage.

```
5' Flanking
Protein   D  S  S  A  T  P  H  L  D  A  V  E  Q  T  L  R  Q  V  S  P  G  L  E  G  R  V  W  E  R  T
Optimized GATAGCTCTGCGACCCCGCACCTGGATGCGGTTGAACAGACCCTGCGTCAGGTTAGCCCTGGTCTGGAAGGCCGCGTTTGGGAACGTACC
Protein   A  G  N  A  L  D  A  P  A  G  D  P  A  G  W  L  L  Q  T  P  G  C  W  G  D  A  N  C  A  E
Optimized GCGGGTAACGCTCTGGATGCGCCGGCGGGTGATCCGGCGGGTTGGCTGTTGCAGACCCCAGGTTGCTGGGGTGATGCGAACTGCGCGGAA
Protein   R  T  G  T  K  R  L  L  A  R  M  T  E  N  I  S  K  A  T  R  T  V  D  I  S  T  L  A  P  F
Optimized CGTACCGGCACCAAACGTCTGCTGGCACGTATGACCGAAAACATCTCTAAAGCGACCCGCACCGTTGACATCTCTACCCTGGCGCCGTTC
Protein   P  N  G  A  F  Q  D  A  I  V  A  G  L  K  K  S  V  E  N  G  N  K  P  K  V  R  V  L  V  G
Optimized CCGAACGGTGCGTTCCAGGATGCGATCGTTGCGGGTCTGAAAAAGTCTGTTGAAAACGGTAACAAACCGAAAGTTCGCGTTCTGGTGGGT
Protein   A  A  P  V  Y  H  M  N  V  L  P  S  K  Y  R  D  D  L  R  D  K  L  G  K  A  A  D  G  L  T
Optimized GCAGCGCCGGTTTACCACATGAACGTTCTGCCGTCCAAATACCGTGATGATCTGCGTGATAAACTGGGCAAAGCCGCGGATGGCCTGACC
Protein   L  N  V  A  S  M  T  T  S  K  T  A  F  S  W  N  H  S  K  L  L  V  V  D  G  Q  S  A  I  T
Optimized CTGAACGTTGCAAGCATGACCACCAGCAAAACCGCATTCAGCTGGAACCACTCTAAACTGCTGGTTGTGGATGGTCAGTCTGCGATCACC
Protein   G  G  I  N  S  W  K  D  D  Y  V  D  T  T  H  P  V  S  D  V  D  L  A  L  T  G  P  A  A  G
Optimized GGTGGCATCAACAGCTGGAAAGATGATTACGTTGACACCACCCACCCGGTGAGCGACGTTGATCTGGCGCTGACCGGTCCGGCGGCGGGT
Protein   S  A  G  R  Y  L  D  Q  L  W  T  W  T  C  E  N  K  S  N  I  A  S  V  W  F  A  A  S  P  G
Optimized AGCGCGGGTCGTTACCTGGACCAGCTGTGGACCTGGACCTGCGAAAACAAATCCAACATCGCAAGCGTTTGGTTTGCGGCCTCTCCGGGC
Protein   A  G  C  M  P  T  M  E  K  D  A  N  P  V  P  A  A  A  T  G  N  V  P  V  I  A  V  G  G  L
Optimized GCTGGCTGTATGCCGACGATGGAAAAAGATGCTAACCCGGTTCCGGCGGCTGCGACCGGTAACGTTCCGGTGATCGCGGTGGGCGGTCTG
Protein   G  V  G  I  K  D  S  D  P  S  S  A  F  K  P  E  L  P  S  A  P  D  T  K  C  V  V  G  L  H
Optimized GGTGTTGGCATCAAAGATTCCGATCCGAGCAGCGCGTTCAAACCGGAACTGCCGAGCGCCCCGGATACCAAATGCGTTGTTGGTCTGCAC
Protein   D  N  T  N  A  D  R  D  Y  D  T  V  N  P  E  E  S  A  L  R  A  L  V  G  S  A  R  S  H  V
Optimized GATAACACCAACGCGGACCGTGATTACGATACCGTTAACCCGGAAGAAAGCGCGCTGCGTGCTCTGGTGGGCAGCGCGCGTTCCCACGTT
Protein   E  I  S  Q  Q  D  L  N  A  T  C  P  P  L  P  R  Y  D  V  R  L  Y  D  A  L  A  A  K  L  A
Optimized GAAATCTCTCAGCAGGATCTGAACGCGACCTGCCCGCCGCTGCCGCGTTATGACGTGCGCCTGTACGATGCACTGGCGGCGAAACTGGCG
Protein   A  G  V  K  V  R  I  V  V  S  D  P  E  N  R  G  A  V  G  S  G  G  Y  S  Q  I  K  S  L  N
Optimized GCTGGCGTGAAAGTGCGTATCGTTGTGAGCGACCCGGAAAACCGTGGCGCGGTTGGCTCTGGCGGTTACTCTCAGATCAAATCCCTGAAC
Protein   E  I  S  D  L  L  R  N  R  L  S  L  L  P  G  G  A  Q  G  A  K  T  A  M  C  G  N  L  Q  L
Optimized GAAATCTCCGACCTGCTGCGTAACCGTCTGAGCCTGCTGCCGGGCGGTGCTCAGGGTGCTAAAACCGCTATGTGCGGTAACCTGCAACTG
Protein   A  T  A  R  S  S  D  S  A  K  W  A  D  G  K  P  Y  A  Q  H  H  K  L  V  S  V  D  D  S  A
Optimized GCGACCGCGCGCAGCAGCGACTCTGCTAAATGGGCTGATGGTAAACCGTACGCGCAGCACCACAAACTGGTTAGCGTTGATGATTCTGCA
Protein   F  Y  I  G  S  K  N  L  Y  P  S  W  L  Q  D  F  G  Y  I  V  E  S  P  E  A  A  R  Q  L  D
Optimized TTCTACATCGGTAGCAAAAACCTGTACCCGAGCTGGCTGCAAGATTTCGGTTACATCGTTGAAAGCCCGGAAGCGGCGCGTCAGCTGGAT
Protein   A  E  L  L  A  P  Q  W  K  Y  S  Q  A  T  A  T  F  D  Y  A  R  G  I  C  Q  G
Optimized GCGGAACTGCTGGCGCCGCAGTGGAAATACAGCCAGGCGACCGCGACCTTCGATTACGCGCGTGGCATCTGCCAGGGT
3' Flanking
```

The PCR conditions were as follows: denaturation at 94 °C for 5 min, followed by 30 cycles of 30 s at 94 °C for denaturation, 30 min at 58 °C for annealing, and 2 min at 72°C for extension. Finally, 10 min at 72°C for extension.
